# Associations between Pro- and Anti-Inflammatory Gastro-Intestinal Microbiota, Diet, and Cognitive Functioning in Dutch Healthy Older Adults: The NU-AGE Study

**DOI:** 10.3390/nu12113471

**Published:** 2020-11-12

**Authors:** Annick P. M. van Soest, Gerben D. A. Hermes, Agnes A. M. Berendsen, Ondine van de Rest, Erwin G. Zoetendal, Susana Fuentes, Aurelia Santoro, Claudio Franceschi, Lisette C. P. G. M. de Groot, Willem M. de Vos

**Affiliations:** 1 Division of Human Nutrition and Health, Wageningen University & Research, 6708WE Wageningen, The Netherlands; agnes.berendsen@wur.nl (A.A.M.B.); ondine.vanderest@wur.nl (O.v.d.R.); lisette.degroot@wur.nl (L.C.P.G.M.d.G.); 2 Laboratory of Microbiology, Wageningen University & Research, 6708WE Wageningen, The Netherlands; gerben.hermes@wur.nl (G.D.A.H.); erwin.zoetendal@wur.nl (E.G.Z.); susana.fuentes@rivm.nl (S.F.); willem.devos@wur.nl (W.M.d.V.); 3 Center for Infectious Disease Control, National Institute for Public Health and the Environment, 3721MA Bilthoven, The Netherlands; 4 Department of Experimental, Diagnostic and Specialty Medicine, University of Bologna, 40126 Bologna, Italy; aurelia.santoro@unibo.it (A.S.); claudio.franceschi@unibo.it (C.F.); 5 AlmaMater Research Institute on Global Challenges and Climate Change (Alma Climate), University of Bologna, 40126 Bologna, Italy; 6 Department of Applied Mathematics, Institute of Information Technology, Mathematics and Mechanics (ITMM), Lobachevsky State University of Nizhny Novgorod-National Research University (UNN), 603950 Nizhny Novgorod, Russia; 7 Human Microbiome Research Program, Faculty of Medicine, University of Helsinki, 00014 Helsinki, Finland

**Keywords:** gut microbiota, dietary intake, cognitive decline, elderly, healthy ageing, inflammation

## Abstract

Dietary modulation of the gastro-intestinal microbiota is a potential target in improving healthy ageing and age-related functional outcomes, including cognitive decline. We explored the association between diet, gastro-intestinal microbiota and cognition in Dutch healthy older adults of the ‘New dietary strategies addressing the specific needs of the elderly population for healthy aging in Europe’ (NU-AGE) study. The microbiota profile of 452 fecal samples from 226 subjects was determined using a 16S ribosomal RNA gene-targeted microarray. Dietary intake was assessed by 7-day food records. Cognitive functioning was measured with an extensive cognitive test battery. We observed a dietary and microbial pro- to anti-inflammatory gradient associated with diets richer in animal- or plant-based foods. Fresh fruits, nuts, seeds and peanuts, red and processed meat and grain products were most strongly associated to microbiota composition. Plant-rich diets containing fresh fruits, nuts, seeds and peanuts were positively correlated with alpha-diversity, various taxa from the Bacteroidetes phylum and anti-inflammatory species, including those related to *Faecalibacterium prausnitzii* and *Eubacterium rectale* and *E. biforme*. Animal product-rich diets associated with pro-inflammatory species, including those related to *Ruminococcus gnavus* and *Collinsella spp*.. Cognition was neither associated with microbiota composition nor alpha-diversity. In conclusion, diets richer in animal- and plant-based foods were related to a pro- and anti-inflammatory microbial profile, while cognition was associated with neither.

## 1. Introduction

The ageing population is growing rapidly. Worldwide, the number of people aged 65 years or over is currently estimated at 703 million. Due to a steep rise in life expectancy, this number is expected to double to 1.5 billion in 2050 [1]. Unfortunately, as the longer lifespan is not accompanied by improvements of health outcomes [2], the increase in life expectancy poses serious challenges to the health care system, economy and society [3]. Therefore, there is an urgent need for strategies to improve healthy ageing.

The gastro-intestinal (GI) microbiota has been implicated as a potential target to enhance healthy ageing [4]. Ageing is accompanied by several physiological and lifestyle changes, including altered GI tract function, elevated inflammation levels and dietary changes, that affect the GI microbiota [5,6]. Compared to younger adults, the GI microbiota in older adults has been shown to exhibit larger inter-individual and temporal variation. It was also strongly correlated to diet, which was linked to residence location in the community [7,8]. Despite the larger variation, several universal changes in the GI microbiota that occur with ageing have been identified. Generally, the relative abundance of *Bifidobacterium* spp. was found to be lower in older adults with concomitant higher levels of Enterobacteriaceae and other pathobionts [5,6].

Changes in GI microbiota composition may influence age-related functional outcomes, such as cognitive decline. In the past decade, the link between altered GI microbiota composition and cognition has been demonstrated in various rodent models, including germ-free animals and several microbiota modulation strategies, such as antibiotics, pre- or pro-biotics, and fecal microbiota transplants [9]. For example, rodents with disrupted GI microbial homeostasis, due to infection or treatment with antibiotics, perform worse on cognitive tests compared to animals with an undisturbed GI microbiota. Restoring this homeostasis by administration of probiotics or via fecal microbiota transplantation positively influenced cognitive performance of rodents [9]. In humans, administration of *Bifidobacterium* and *Lactobacillus* species for 12 weeks has shown to positively affect cognitive functioning in older adults [10,11], providing preliminary evidence for a relation between GI microbiota and cognition in humans, thus proposing the GI microbiota as a target to prevent or delay age-related cognitive decline.

Modification of diet has been suggested as a strategy to both maintain cognition and GI homeostasis. There is special interest in the Mediterranean diet (MedDiet), which is characterized by a high intake of vegetables, fruits, legumes and olive oil and moderate to low intake of animal-based food products [12]. Greater adherence to the MedDiet has been associated to slower rates of age-related cognitive decline [13,14] and beneficial changes in GI microbiota composition [15,16]. 

To our knowledge, to date only one human study has investigated the relation between diet, cognition and GI microbiota. Data from all European partners of the ‘New dietary strategies addressing the specific needs of the elderly population for healthy aging in Europe’ (NU-AGE) study, a one-year Mediterranean-like dietary intervention, showed that individuals with better adherence to this diet had higher relative abundances of several microbial groups, including *Faecalibacterium prausnitzii, Anaerostipes* and *Roseburia* [16], which have previously been linked to beneficial health effects. For instance, these species exhibit anti-inflammatory properties, are able to produce the short chain fatty acid (SCFA) butyrate and have been inversely associated with diabetes mellitus type 2 and colorectal cancer [17,18,19]. In turn, higher relative abundances of these beneficial species were weakly, but positively, associated with cognitive function measured by BabCock memory and constructional praxis performance [16]. 

These results provide preliminary evidence for the potential of the MedDiet to prevent age-related cognitive decline by modulating GI microbiota. However, it remains unclear which specific food groups of the MedDiet are responsible for the potentially beneficial effects on cognition and GI microbiota composition. Moreover, in the previous study, cognitive function was measured by means of single tests [16], whereas the assessment of multiple cognitive tests representing all cognitive domains and combining these tests into composite cognitive scores is a more robust measure of cognitive functioning [20]. Therefore, the current study aims to explore the relation between diet, GI microbiota composition and cognitive function in healthy older adults (65–79 years). 

## 2. Materials and Methods 

### 2.1. Study Design and Participants

We used data from the Dutch cohort of the NU-AGE study, a parallel randomized one-year study investigating the effect of a dietary intervention on inflammation in European older adults [21]. Cognitive functioning and microbiota composition were determined as secondary outcomes. Information on participants, recruitment and the dietary intervention has previously been described in detail [22,23]. In short, 252 healthy Dutch older adults aged 65–79 years were randomized to the intervention or control group. Participants in the intervention group received individually tailored dietary advice to follow a Mediterranean-like diet. The control group received no specific dietary advice except for a leaflet describing the national guidelines for a healthy diet. Analyses showed that the intervention did not affect GI microbiota. Therefore, the current study has a cross-sectional design, in which data from both pre and post intervention are combined. Participants were non-frail (fried frailty ≤ 1 [24]) and free of major diseases including cancer, dementia, diabetes mellitus type I and II and organ failure, and did not use antibiotics in the three months prior to inclusion. Dietary intake, GI microbiota composition and cognitive functioning were assessed at baseline and post intervention. Data from 26 participants were excluded due to missing GI microbiota assessments at either pre or post intervention. The NU-AGE study has been registered at clinicaltrials.gov (identifier: NCT01754012). This study was conducted according to the Declaration of Helsinki and written informed consent was obtained from all participants. The study protocol was approved by the Medical Ethics Committee of Wageningen University and Research (ABR 37818.081.11).

### 2.2. Dietary Assessment

At baseline and post intervention, dietary intake was assessed by a 7-day food record. Participants were instructed to record all consumed foods and their amounts based on household measures. All food records were reviewed by a trained research dietician during an interview. Consumed food products were coded according to standardized coding procedures. Nutrient intake data was calculated by use of the Dutch food composition table (NEVO 2011). Consumed food products with similar composition were grouped into food groups according to the EPIC-Soft Classification [25] with some local modifications. Additional groups were created for ready-to-eat meals and savory bread spreads as products in these groups were not included in the current EPIC-Soft list. Separate groups were created for low fat, and salt and sugar options within the dairy food groups based on the Dutch dietary guidelines [26]. Products containing artificial sweeteners were placed in a separate group as sweeteners have been shown to influence GI microbiota composition [27]. In addition, a separate group was made for legume-based ready to eat soups due to the relatively high fiber content. Finally, the food group meat was divided into red meat, processed meat, poultry and meat replacers instead of groups based on animal origin to limit the number of food groups.

### 2.3. Microbiota Composition Profiling

At baseline and post intervention, participants were instructed to collect a fecal sample at home with the help of a stool collection kit and store them immediately at −20 °C. Samples were transported in coolers and then stored at −20 °C and later at −80 °C before being processed. DNA extraction from fecal samples has been described in detail elsewhere [28]. In brief, DNA was extracted using a combination of column purification and repeated-bead-beating. Purity and concentration of DNA were assessed with a Nanodrop 1000 spectrophotometer (Thermo Fisher Scientific, Wilmington, USA). The composition analysis was then performed utilizing a previously benchmarked custom made, phylogenetic microarray, the human intestinal tract chip (HITChip) [29,30]. The HITChip contains a duplicated set of 3631 probes, which target the V1 and V6 hypervariable regions of the 16S rRNA gene of 1140 intestinal bacterial phylotypes. After extraction of DNA, the full-length 16S rRNA gene was amplified by PCR using primers T7prom-Bact-27-for and Uni-1492-rev [30]. This was followed by in vitro transcription and labelling of the resulting RNA with Cy3/Cy5 before hybridization to the array. The signal intensity data from the microarray hybridizations were collected from the Agilent G2505C scanner (Agilent Technologies) using Agilent Feature Extraction software, version 10.7.3.1 and pre-processed using an in-house MySQL database and custom R scripts. Each scanner channel from the array was separately spatially normalized using polynomial regression, followed by outlier detection and filtering in each set of probes with a χ2 test. Each sample was hybridized at least twice to ensure reproducibility. Duplicate hybridizations with a Pearson correlation < 0.98 were not considered for further analysis. Microbiota profiles were summarized to genus-like 16S rRNA gene sequence groups with a sequence similarity > 90% referred to as species and relatives (‘et rel.’). Measurements of probes that belong to the same phylotype were normalized with robust probabilistic averaging [31,32]. Log10-transformed hybridization signals were used as a proxy for bacterial abundance.

### 2.4. Cognitive Functioning

Cognitive functioning was assessed at baseline and post intervention with an extensive battery of cognitive tests which were administered by trained research assistants. The battery included cognitive tests from the consortium to establish a registry for Alzheimer’s disease (CERAD) test battery [33] plus five additional tests.

In the verbal fluency category test [34], participants were asked to name as many animals as possible within 60 s. The number of uniquely named animals was recorded. Participants were presented with four figures in the constructional praxis test [35], and asked to copy these figures on blank paper immediately after presentation (subtest immediate) and after a few minutes (subtest recall). Scoring was based on the number of correct responses. In the word list memory test [33], participants were visually presented with ten random words. The number of correctly recalled words directly after presentation in three trials (subtest immediate) and after five minutes in one trial (subtest delayed) was recorded. Finally, the participant was asked to identify the ten words from a verbally presented list of twenty words (subtest recognition). Next, participants were read a brief story in the Babcock story recall test [36] and asked to retell the story immediately (subtest immediate) and after 20 min (subtest delayed). Scoring was based on the correctly recalled parts of the story. In the trail making test [37], participants were instructed to connect 25 numbers in chronological order (Part A) and to connect numbers and letters in chronological and alphabetical order alternately (Part B). Time to complete each task was recorded. In the number cancellation test [38], participants were presented with a list of random numbers. The number of correctly crossed out 4s in 30 s was documented. In the pattern comparison test [39], participants were asked to indicate if two patterns were similar or different. Scoring was based on the number of correct responses.

Scores for each of the cognitive tests were converted into Z-scores with baseline mean and standard deviation of the whole population. The Z-score for the trail making test was reversed as lower scores represent better cognitive functioning. The individual Z-scores for the cognitive tests were clustered into four cognitive domains:

Episodic memory = (zWordList_immediate_ + zWordList_delayed_ + zWordList_recognition_ + zBabcockStoryRecall_immediate_ + zBabcockStoryRecall_delayed_)/5 (1)

Executive functioning = (zVerbalFluency + -zTrailMakingTest_B/A_) /2 (2)

Information processing speed = (-zTrailMakingTest_A_ + zNumberCancellation + zPatternComparison)/3 (3)

Visuospatial ability = (zConstructionalPraxis_immediate_ + zConstructionalPraxis_recall_)/2 (4)

### 2.5. Assessment of Phenotypical Characteristics

Body weight and height were measured by trained research assistants. Weight was determined while wearing light clothing to the nearest 0.1 kg using a calibrated scale. Height was measured using a stadiometer to the nearest 0.1 cm. Body mass index (BMI) was calculated as weight/height^2^. Data on age, sex, education (number of years) and smoking status (never, former or current) were collected using questionnaires. Frailty status (non-frail/pre-frail) [24] and mini-mental state examination (MMSE) [40] were assessed by trained research assistants following standardized procedures. MMSE scores from 24 to 30 are considered within the normal range [40]. Physical activity was measured using the physical activity scale for elderly (PASE). For individuals aged 70 to 75, average values for PASE are 89.1 for women and 102.4 for men [41]. 

### 2.6. Statistical Analyses

All microbiota analyses were performed in R version 3.4.0 [42]. Redundancy analysis (RDA) was performed to determine the multivariate effects of the explanatory variables on microbiota composition using the rda function from the vegan package [43]. RDA is a technique summarizing the linear relationships between a set of variables i.e., GI microbiota composition explained by a set of explanatory variables, i.e., dietary and host variables. The effect of an explanatory variable is defined as R^2^, which is the percentage of variation explained from the total amount of microbiota variation. All numerical environmental variables (food groups, nutrients, phenotype and cognition) were normalized to ensure that the input variables had similar scales before performing the RDA. We first determined the simple effects of all explanatory variables on microbiota composition to help understand what was driving the interactions. Because the dietary intervention had no significant effect on microbiota composition, we performed a cross-sectional analysis with both pre- and post-intervention samples to increase power. To determine which set of food groups resulted in the most parsimonious model (i.e., explaining microbiota variation), we performed forward and reverse automatic stepwise model selection for constrained ordination methods using permutation tests with the ordistep function from the vegan package, which bases the term choice on Akaike’s information criterion and *p*-value. This ordination configuration was used to test which other explanatory variables (nutrients, phenotype and cognition) significantly correlated with microbiota composition by post-hoc fitting these as vectors using the envfit function from vegan. Significance was set at *p* < 0.05. Richness, inverse Simpson and Shannon diversity were calculated to define microbial alpha-diversity using the microbiome package [44]. In ecology, alpha-diversity is defined as the species diversity within a sample. We used to commonly applied methods to determine diversity, *viz* Shannon diversity and inverse Simpson diversity. Diversity of the microbiota was based on non-logarithmic oligo-level signals and probes were counted in each sample to measure richness, by using an 80% quantile threshold for detection. To correlate microbial alpha-diversity with the significant explanatory variables we used Pearson correlations and visualized these using heatmaps with the psych package [45]. *p*-values were corrected for multiple testing using the Benjamini–Hochberg procedure [46] and q < 0.05 was considered significant.

## 3. Results

### 3.1. Participant Characteristics

At baseline, the mean age of participants was 70.9 ± 4.1 years and 44.4% of the study population was male (Table 1). The average body mass index (BMI) at baseline was 25.9 ± 3.6 kg/m^2^ and mean score on the mini-mental state examination (MMSE) was 27.7 ± 1.8 points, indicating that our study population was cognitively healthy. The mean PASE score was 137 ± 54, indicating that the physical activity level was slightly higher than normal compared to a study population with similar age [41]. 

### 3.2. Variables Affecting GI Microbiota Composition

**Figure 1:**
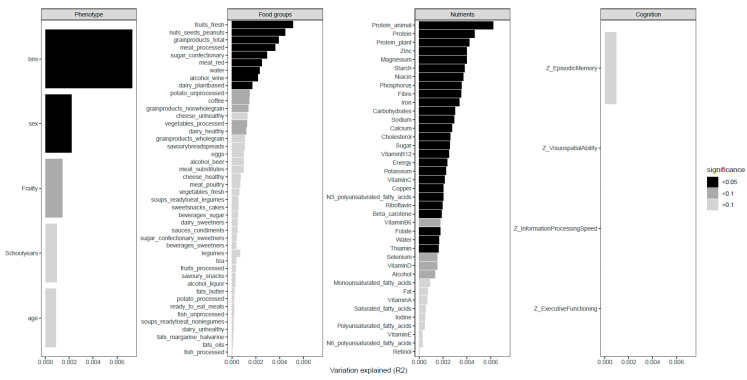
Microbiota covariates. Impact of all measured variables on microbiota composition defined as percentage variation explained (R^2^) out of all the total microbiota variation. A higher R^2^ implies a stronger effect size.

To determine how the different environmental variables impact the microbiota, we first calculated their simple effects (i.e., the effect of the environmental variable on the microbiota without any other covariates). As previously described in the methods, the dietary intervention had no significant effect on microbiota composition (*p* = 1.0, R^2^ = 0.08%). A total of 41 variables, existing of phenotypical characteristics, food groups and nutrients, significantly correlated to GI microbiota composition as shown in Figure 1. The largest proportion of GI microbiota variation was explained by individuals (R^2^ = 40.0%) (Supplementary Figure S1). The phenotypical characteristics BMI (R^2^ = 0.73%) and sex (R^2^ = 0.22%) were both correlated with microbiota composition. BMI explained the largest proportion of microbiota variation out of all microbiota covariates. With respect to the dietary variables, 29 nutrients and 10 food groups were significantly correlated with GI microbiota composition. Concerning the food groups, fresh fruits explained the highest proportion of variation in GI microbiota composition (R^2^ = 0.51%). Further zooming in on the fresh fruits showed that berries and grapes were the fruits most contributing to this observation. Other significant food groups were nuts, seeds and peanuts (R^2^ = 0.45%), grain products (R^2^ = 0.39%) and both processed and red meat (R^2^ = 0.36% and R^2^ = 0.25% respectively). Among the nutrients, total protein (R^2^ = 0.46%) and protein from animal (R^2^ = 0.62%) and plant (R^2^ = 0.42%) sources explained the largest proportion of variation. In addition, various forms of carbohydrates, water-soluble vitamins, minerals and omega-3 fatty acids were significantly associated to GI microbiota composition, while other fatty acids and fat-soluble vitamins did not. None of the cognitive functioning domains was significantly correlated with GI microbiota composition.

**Figure 2:**
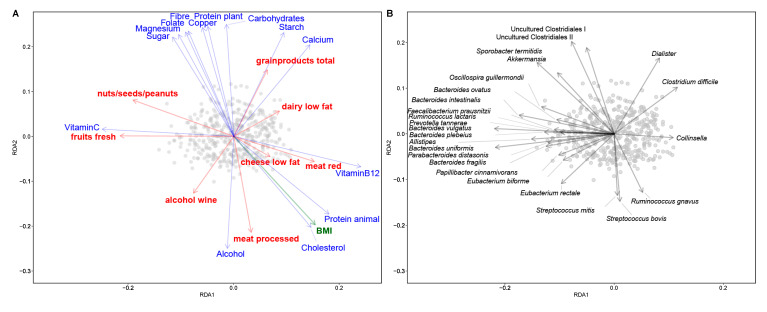
Association of microbiota with food groups, nutrients and BMI. Samples are plotted as grey circles. (**A**) Redundancy analysis (RDA) bi-plot of microbiota with explanatory variables; food groups (red), nutrients (blue) and phenotypical characteristics (green). (**B**) RDA bi-plot of samples with the associated microbial taxa (indicated as genera or species-level groups) The direction of the arrows depicts the abundance of microbial taxa. Length of the arrows is a measure of fit. The variable arrows approximate the correlation between species and explanatory variables. Samples near the coordinate origin (zero point) suggest near zero correlation. The further a sample falls in the direction indicated by the arrow, the higher the correlation.

To visualize the relations between dietary factors and phenotypical characteristics with microbiota composition, their conditional effects (the impact on the microbiota with the effect of other variables in the model) were calculated and plotted in two RDA bi-plots (Figure 2). We observed a gradient of participants with higher intakes of plant-based foods and participants consuming higher amounts of animal-based foods. Higher intakes of these animal-based foods, animal protein, cholesterol, vitamin B12, low fat cheese, and red and processed meat, were correlated with a higher BMI. The participants with lower intake of animal-based foods and higher intake of plant-based foods could be further divided into two groups; those consuming higher amounts of fresh fruits, nuts, seeds and peanuts and vitamin C, and those with higher intakes of grain products and digestible carbohydrates. 

Consumption of animal-based foods and BMI was positively associated with species related to *Ruminococcus gnavus, Streptococcus* spp. (*S. mitis* and *bovis*) and *Collinsella*. Conversely, animal-based foods were inversely associated with *Akkermansia muciniphila,* uncultured Clostridiales I and II and species related to *Sporobacter termitidis.* Consumption of fresh fruits, its associated nutrient vitamin C, and nuts, seeds and peanuts were associated with several genera from the Bacteroidetes phylum, including *Bacteroides* spp., *Parabacteroides*, *Alistipes* and *Prevotella,* and Firmicutes such as species related to *Faecalibacterium prausnitzii, Oscillospira guillermondii* and *Eubacterium rectale* and *E. biforme.* Grain products and carbohydrates were positively associated with *Dialister* and species related to *Clostridium difficile* (recently renamed to *Clostridioides difficile*). Although this group is named after *C. difficile*, the observed differences do likely not relate to this potential pathogen but probes targeting *C. bifermentans*, *C. bartlettii* and *C. glycolicum.*

### 3.3. Variables Associated with Microbial Alpha-Diversity

**Figure 3:**
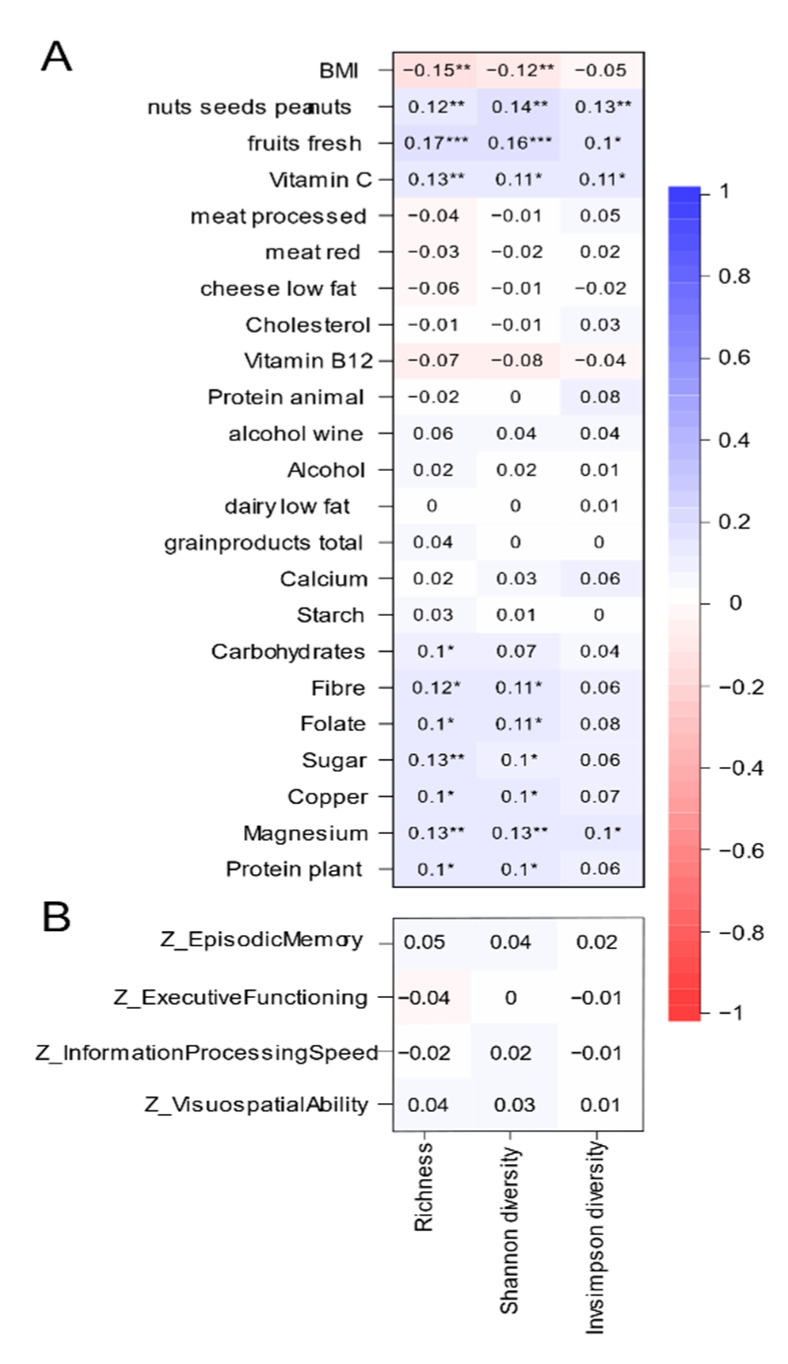
Correlation of alpha-diversity with microbiota covariates (**A**) and cognition variables (**B**). Pearson correlation of significant microbiota covariates were calculated. *p* values are corrected for multiple testing using the Benjamini–Hochberg procedure. *** q < 0.001, ** q < 0.01 * q < 0.05.

The relations between the significant variables in the RDA (phenotypical characteristics, nutrients, food groups) and indices that contribute to microbial alpha-diversity were calculated and visualized in Figure 3A. BMI was negatively correlated with alpha-diversity. With respect to the food groups, only fresh fruits and nuts, seeds and peanuts were positively correlated with alpha-diversity, with correlation coefficients ranging from 0.1 to 0.17. Among the fresh fruits, alpha diversity positively correlated with berries and grapes, citrus fruits and stone fruits in Supplementary Table S1. Nutrients that were positively correlated to alpha-diversity included vitamin C, various minerals, forms of carbohydrate and plant protein, with correlation coefficients between 0.09 and 0.14. None of the nutrients was negatively associated with alpha-diversity. 

With correlation coefficients ranging from −0.04 to 0.05, none of the cognitive domains was significantly correlated to any of the diversity indices (Figure 3B). 

## 4. Discussions

By exploring associations between diet, GI microbiota and cognition in healthy Dutch older adults using food groups as the primary input, we showed that fresh fruits, nuts, seeds and peanuts, red and processed meat, grain products, low fat dairy and cheese and wine are important dietary factors in GI microbiota composition. Of these food groups, fresh fruits (berries and grapes in particular), and nuts, seeds and peanuts positively correlated with alpha-diversity. Overall, fresh fruits and nut seeds and peanuts correlated with various taxa from the Bacteroidetes phylum and species related to *Faecalibacerium prausnitzii*, grain products correlated with *Dialister,* while higher intake of animal-based foods was associated with a higher abundance of *Collinsella* and *Streptococcus* spp. as well as species related to *Ruminococcus gnavus*. Cognitive functioning was neither associated with GI microbiota composition nor alpha-diversity. 

Our study is the first to investigate which food groups are related to whole GI microbiota composition and alpha-diversity in older adults. In younger adults, several studies have investigated this association before. In a large cross-sectional study with GI microbiota data from 1135 Dutch adults, 78 dietary factors, including fruit, frequency of nut consumption, red and processed meat and protein, were important dietary factors in explaining GI microbiota variation [47]. The associations of fruit and meat with GI microbiota composition were confirmed in a large cross-sectional Belgian study with adults (*n* = 1106) [48] and the French Milieu Intérieur study (*n* = 862) [49] showed that fruit influenced the GI microbiota. With respect to alpha-diversity, our finding that individuals with higher intakes of fresh fruit and nuts had a more diverse GI microbiota was confirmed by the studies of Dutch and French adults [47,49] and the association between nuts, seeds and peanuts and alpha-diversity was also observed in Dutch adults [47]. 

Despite the fact that the dietary intervention did not have a significant impact on the GI microbiota in our cohort, we could clearly identify associations between dietary variables and microbiota composition. We observed a gradient between participants consuming a diet richer in foods from animal origin and a diet richer in foods from plant origin, from now on referred to as animal- and plant-rich diets. The animal-rich diet was characterized by higher intakes of processed and red meat, low fat cheese and dairy, vitamin B12 and cholesterol. The plant-rich diet was higher in vitamin C, fresh fruits and nuts, seeds and peanuts. In addition to the classification based on origin of the food products and nutrients, these diets can also be classified as pro- and anti-inflammatory according to the dietary inflammatory index, in which various dietary factors have been scored based on their inflammatory potential [50]. Vitamin B12 and cholesterol, both associated with the animal-rich diet, were considered pro-inflammatory. With respect to the plant-rich diet, nutrients present in fresh fruits (vitamin C, flavonoids, fiber) and nuts, seeds and peanuts (polyphenols, omega-3 fatty acids, fiber) were all classified as anti-inflammatory.

Interestingly, classification of the GI microbiota based on inflammatory potential showed a similar pattern. The consumption of the pro-inflammatory diet rich in animal foods positively correlated with *Collinsella* and *Streptococcus* spp. as well as species related to *R. gnavus*. Overall, these bacteria have been classified as pro-inflammatory. Increased abundance of *Collinsella* has been observed in several inflammatory diseases, including type 2 diabetes mellitus [51,52], atherosclerosis [53] and rheumatoid arthritis [54]. Even though *Streptococcus* is a normal inhabitant of the upper GI tract, increased abundance in the colon has been associated with pro-inflammatory nutrients of animal origin [15]. Finally, higher abundance of *R. gnavus* has been linked to several inflammatory diseases as well, such as spondyloarthritis [55], eczema in infants [56] and inflammatory bowel disease, especially during active disease episodes [57]. In addition to the connection with inflammatory diseases, it has recently been shown that *R. gnavus* synthesizes an inflammatory polysaccharide that induces secretion of the inflammatory cytokine tumor necrosis factor-alpha by dendritic cells [58].

The anti-inflammatory plant-rich diet was associated with species related to *F. prausnitzii*, *E. rectale* and *E. biforme*. These species can be classified as anti-inflammatory due to their ability to produce butyrate. Butyrate has been shown to exhibit anti-inflammatory effects through their regulation of leukocyte function via inhibition of histone deacetylase and activation of G-protein coupled receptors [59]. These anti-inflammatory effects of butyrate have been demonstrated in vivo, in both animal models [60] and human clinical trials [61]. *F. prausnitzii* specifically has been shown to exhibit anti-inflammatory effects in vitro and in vivo. In peripheral blood mononuclear cells, *F. prausnitzii* led to higher levels of the anti-inflammatory cytokine IL-10 and lower production of the pro-inflammatory cytokines IL-12 and IFN-γ. In a mouse model with induced acute colitis, administration of living *F. prausnitzii* decreased colitis [62]. Moreover, in humans lower abundance of these species has been observed in several inflammatory diseases. A meta-analysis in inflammatory bowel disease patients showed that patients suffering from an active disease episode had lower abundance of *F. prausnitzii* compared to patients in remission [63] and *E. rectale* were reduced in Crohn’s disease patients compared to healthy controls [64]. The plant-rich diet also positively correlated to the mucin degrading species *A. muciniphila*. Similarly, lower abundance of *A. muciniphila* has been observed in inflammatory conditions including obesity and type 2 diabetes [65,66,67]. Moreover, a recent human intervention trial showed that daily administration of *A. muciniphila* cells for three months increased barrier function, by decreasing the levels of pro-inflammatory lipopolysaccharides in prediabetic human subjects [68]. Overall, the links between these bacteria and inflammatory diseases and compounds, indicate that the consumption of an animal-rich diet might correlate with a more pro-inflammatory GI microbiota profile, while the plant-rich diet correlates to a more anti-inflammatory GI microbiota profile. 

Moreover, several species associated to the plant-rich diet, including *F. prausnitzii* and *E. rectale*, have been previously associated with a high adherence to the MedDiet in various European countries [16]. This might imply that certain food groups that were part of the plant-rich diet, i.e., nuts, seeds and peanuts and fresh fruits, are important dietary factors in the MedDiet with respect to GI microbiota modulation. The beneficial associations of these food groups could be due to the fiber present in fruits and nuts. Fermentation of fibers in the gut leads to the production of SCFA, which have beneficial effects on health as previously discussed [69]. An additional factor underlying the beneficial associations might be the presence of polyphenols in fruit and nuts. These plant metabolites are poorly absorbed in the small intestine and reach the colon where they can interact with microbiota. Polyphenols have been shown to have prebiotic-like effects. Various types of polyphenols enhanced growth of lactobacilli and bifidobacteria as well as *Akkermansia*, in both in vitro and in vivo (animal and human) studies [70,71]. 

In addition to the association between the plant-rich diet and the anti-inflammatory species, the diet rich in plant foods also positively correlated with several genera from the Bacteroidetes phylum such as *Parabacteroides*, *Alistipes*, and mostly *Bacteroides* and *Prevotella* spp.. Members of the latter two maintain a complex and generally beneficial relationship with the host. Bacteroidetes are abundantly present in the human gut and many genera within this phylum respond to changes in diet. Generally, diets rich in fiber are linked with increased abundance of *Prevotella* spp. [72], while higher abundance of *Bacteroides* spp. is associated to diets rich in fat and protein from animal origin [73]. However, the latter group has also been linked to plant-based complex carbohydrates and inversely associated with dietary fat and protein [15], in line with our results. It is well known that microorganisms have context-dependent functions and a changing metabolism, depending on environmental conditions and the presence and function of other microbes. For instance, *Bacteroides* spp. contain a large repertoire of enzymes to break down complex plant carbohydrates [74], which likely underlies their association in the current study. However, several *Bacteroides* spp. are also bile resistant [75] and could thus be more prevalent in individuals consuming high fat diets with little complex carbohydrates. Additionally, different species or strains within the *Bacteroides* and *Prevotella* genera have been shown to be genetically diverse and associated with different dietary components, such as plant-based diets, while some are associated with animal-based nutrients [76,77]. Another factor in the ambiguity of the health associations of *Bacteroides* spp. is their status as a pathogen, as several species (notably *B. fragilis*) can cause significant pathology, including bacteremia and abscess formation in multiple body sites [75]. Similarly, several *Prevotella* spp. have been associated with chronic inflammatory conditions [78]. In contrast, *Bacteroides* spp. have also been linked to beneficial effects on health. This apparent duality was exemplified by the observation of a cohort specific positive or negative association with markers of insulin resistance in overweight insulin resistant males [79]. For example, *Bacteroides* spp. can contribute to the formation the SCFA propionate via the succinate pathway [80]. Propionate has been linked to several health benefits, including regulation of appetite and lipid synthesis in in vivo animal studies, and anti-colorectal cancer effects in in vitro models [81].

Specific food groups, such as berries and nuts, seeds and peanuts, were correlated with several anti-inflammatory microbial species. In addition, these food groups have been associated with slower rates of cognitive decline [82,83]. Although inflammation is a major mechanism underlying cognitive decline [84], we did not find associations between cognitive functioning and the GI microbiota composition or alpha-diversity. To our knowledge, the association between diet, gastro-intestinal microbiota and cognitive functioning in humans has only been investigated in a single other study [16]. Here, the authors showed that European individuals with high adherence to a Mediterranean-like diet had high relative abundance of several beneficial, anti-inflammatory, butyrate producing microbial groups, including *Faecalibacterium prausnitzii, Anaerostipes* and *Roseburia.* Increased relative abundance of these species was associated with improved cognitive function measured by single tests. Our approach augments this paper, but also differed in two aspects. First, we used diet as a combination of different food groups, while in the previous paper diet was only considered as adherence to the Mediterranean diet in general. Hence, it was not clear which specific food groups of the Mediterranean diet were responsible for the beneficial effect on cognition and gastro-intestinal microbiota composition. Second, we incorporated cognitive functioning outcomes using a robust measure of cognitive functioning by calculating mean scores per cognitive domain (composite cognitive scores). The previous research only considered scores of single cognitive tests. Aside from the use of a more robust measure of cognitive functioning, there are several other explanations for the apparent differing results with regard to the association of microbiota with cognitive function.

From animal studies, there is strong evidence for a relation between the gut and the brain, which has been shown with (germ-free) rodent studies, using microbiota modulating strategies such as antibiotics and fecal microbiota transplants [9]. However, there are many differences between rodents and humans, such as differences in GI tract anatomy and physiology and microbiota composition [85], severely limiting translation from rodents to humans. In addition, rodent models allow for more extreme interventions, have a very homogeneous genetic background and there is a high level of control over external factors, which allow for the demonstration of subtle effects. In contrast, we investigated cross-sectional relations in a healthy population of older adults in which diets and microbiota were relatively homogeneous. There were no extreme variations in intake of food components between participants and the dietary intervention that half of the participants underwent, resulted in small changes in dietary intake (e.g., increase of one slice of whole-wheat bread, one third of an apple, and half a serving spoon of vegetables extra per day) [23]. This may have limited the demonstration of associations between cognitive functioning and GI microbiota. 

Moreover, our study population consisted of cognitively healthy older adults as shown by the mean MMSE score of 27.7 points out of 30, as scores from 24 to 30 are considered within the normal range [40]. Cognitively healthy indicates that these participants were no mild cognitive impairment or dementia patients. It is important to emphasize that cognitively healthy older individuals can benefit from the effects of diet on cognition. Cognitive health is not static, but rather a progressive phenomenon. The process of age-related cognitive decline starts from the late 20s and continuous throughout the lifespan [86]. The rate of decline can be influenced by several lifestyle factors, including nutrition. Previous research has already demonstrated that several dietary patterns can slow down cognitive decline with ageing. For example, this has been shown for the Mediterranean, Dietary Approaches to Stop Hypertension (DASH) and Mediterranean-DASH Intervention for Neurodegenerative Delay (MIND) diets [87].

Nevertheless, gastro-intestinal microbiota targeted interventions to slow down cognitive decline may be more effective in cognitively impaired individuals, i.e., mild cognitive impairment or Alzheimer’s disease patients. Mild cognitive impairment and Alzheimer’s disease patients have shown decreased microbial diversity and similar changes in GI microbiota compared to healthy older adults [88]. In line with this, the effectiveness of probiotic supplementation on cognition in humans likely depends on the degree of cognitive impairment. In human intervention studies, the effect of probiotic supplementation on cognitive functioning is mainly effective in cognitively impaired individuals (i.e., mild cognitive impairment or Alzheimer’s disease patients), [10,89,90] while the effectiveness in relatively healthy older adults has been inconsistent [11,91,92]. Similarly, the efficacy of other dietary interventions to slow down cognitive decline has been shown to be dependent on the extent of cognitive impairment as well [93]. Therefore, our study population might have been too healthy to demonstrate the link between cognition and GI microbiota. Indeed, changes in GI microbiota in older adults seem to be more strongly associated with health status rather than with chronological age [5,94]. 

The study population is an important limitation of this study. We did not demonstrate associations between cognitive functioning and GI microbiota, possibly due to relatively small differences in diet and microbiota between subjects and the high cognitive health status of our study population. Further research on the association between diet, GI microbiota and cognitive ageing in humans would benefit from focusing on cognitively impaired study populations and study populations that are more heterogeneous with respect to dietary intake.

## 5. Conclusions

This cross-sectional investigation into the association between diet, GI microbiota and cognition showed that the anti-inflammatory potential of a plant-rich diet high in fresh fruits and nuts, seeds and peanuts was linked to a GI microbiota profile with a higher anti-inflammatory potential. Conversely, a pro-inflammatory animal-rich diet was associated with a more pro-inflammatory GI microbiota profile. Despite the prominent role of inflammation in cognitive decline, we did not demonstrate associations between cognitive functioning and GI microbiota. 

## Figures and Tables

**Table 1:** Baseline characteristics of 226 healthy Dutch older adults.

Characteristic	*n* = 226
Age, years	70.9 ± 4.1
Sex, male *n* (%)	100 (44.2%)
Education, years	12.3 ± 3.7
BMI, kg/m^2^	25.9 ± 3.6
Smoking status, *n* (%)	
Never	117 (51.8%)
Former	103 (45.6%)
Current	6 (2.7%)
MMSE (score 0-30)	27.7 ± 1.8
Physical activity (PASE score)	137 ± 54
Frailty, *n* (%)	
Non-frail	178 (78.8%)
Pre-frail	48 (21.2%)

Abbreviations: BMI: body mass index; MMSE: mini mental state examination; PASE: physical activity scale for the elderly. Data are presented as mean ± SD or number (%).

## Supplementary Materials

The following are available online at https://www.mdpi.com/2072-6643/12/11/3471/s1, Figure S1: microbiota covariates. Impact of the individual and all measured variables and on microbiota composition defined as percentage variation explained (R2) out of the total microbiota variation. Table S1: Pearson correlation of alpha-diversity with fresh fruit categories.
