# Supplementary material for: Associations between Pro- and Anti-Inflammatory Gastro-Intestinal Microbiota, Diet, and Cognitive Functioning in Dutch Healthy Older Adults: The NU-AGE Study
Source: Nutrients. 2020 Nov 12;12(11):3471. doi: 10.3390/nu12113471 (PMC7697493; doi:10.3390/nu12113471)
Supplement: Supplementary file 1 [file nutrients-12-03471-s001.pdf]

Pro- and anti-inflammatory gastro-intestinal microbiota profiles are related to dietary patterns richer in animal- or plant-based foods, but not to cognitive functioning, in Dutch healthy older adults: the NU-AGE study

Annick P.M. van Soest, Gerben D.A. Hermes, Agnes A.M. Berendsen, Ondine van de Rest, Erwin G. Zoetendal, Susana Fuentes, Aurelia Santoro, Claudio Franceschi, Lisette C.P.G.M. de Groot, Willem M. de Vos

Online Supplementary Material

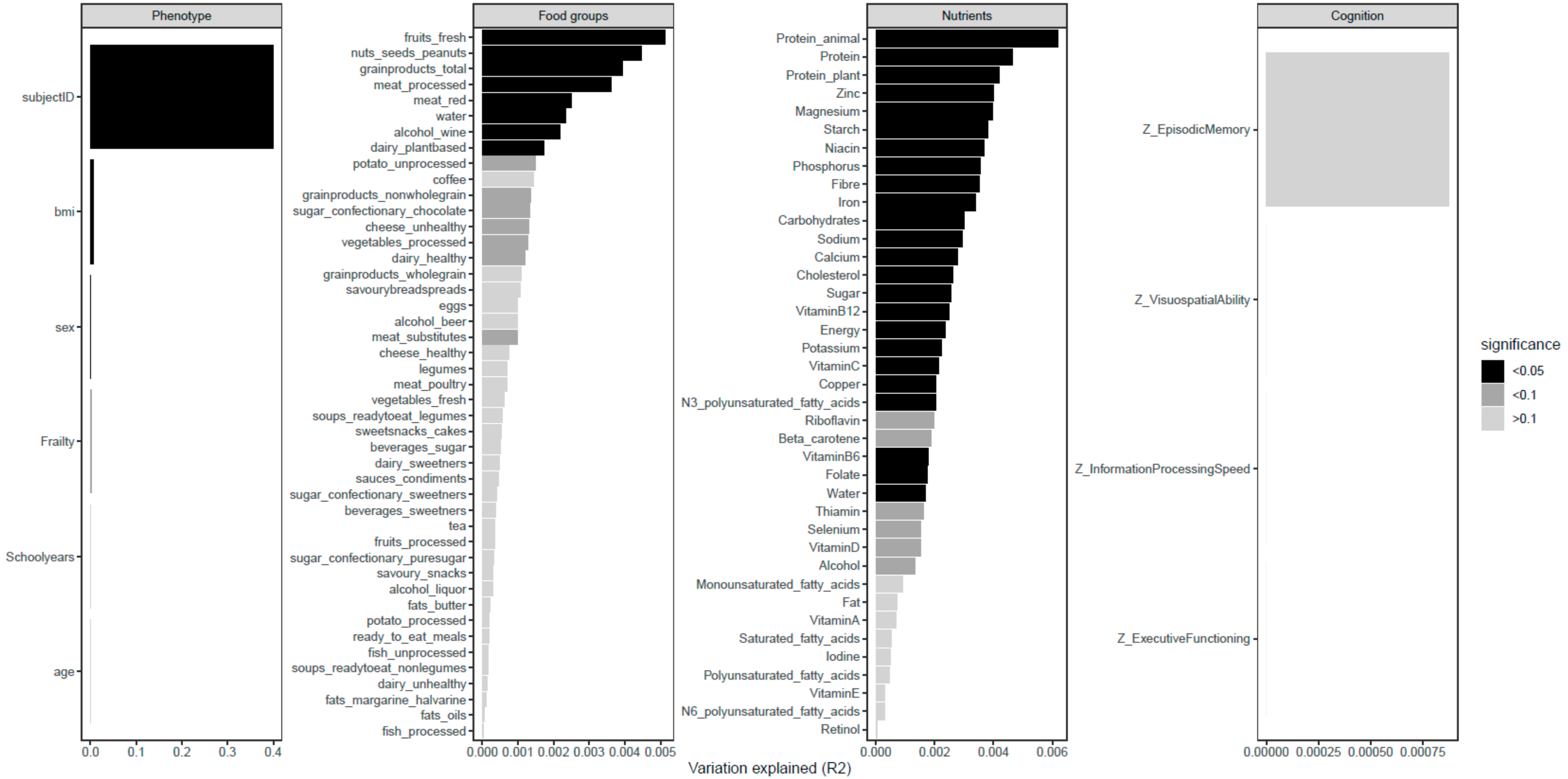

Supplementary figure 1: **Microbiota covariates.** Impact of the individual and all measured variables and on microbiota composition defined as percentage variation explained (R^2) out of the total microbiota variation. A higher R^2 implies a stronger effect size.

Pro- and anti-inflammatory gastro-intestinal microbiota profiles are related to dietary patterns richer in animal- or plant-based foods, but not to cognitive functioning, in Dutch healthy older adults: the NU-AGE study

Annick P.M. van Soest, Gerben D.A. Hermes, Agnes A.M. Berendsen, Ondine van de Rest, Erwin G. Zoetendal, Susana Fuentes, Aurelia Santoro, Claudio Franceschi, Lisette C.P.G.M. de Groot, Willem M. de Vos

Online Supplementary Material

Supplementary table 1. Pearson correlation of alpha-diversity with fresh fruit categories. P-values are corrected for multiple testing using the Benjamini-Hochberg procedure.

	**Richness**	**Corrected p-value**	**Shannon diversity**	**Corrected p-value**	**Inverse Simpson diversity**	**Corrected p-value**
Total fresh fruits	0.170	<0.001	0.157	<0.001	0.105	0.026
Citrus fruits	0.133	0.005	0.105	0.025	0.086	0.067
Apples and pears	0.098	0.037	0.071	0.129	0.022	0.640
Berries and grapes	0.130	0.006	0.115	0.015	0.102	0.030
Banana	-0.065	0.169	-0.048	0.309	-0.092	0.051
Stone fruits	0.062	0.192	0.085	0.071	0.095	0.044
Melon	-0.010	0.827	-0.008	0.867	-0.019	0.692
Tropical fruits	0.086	0.067	0.087	0.063	0.055	0.246
